# Cognitive and Behavioral Disorders in Children with Neurofibromatosis Type 1

**DOI:** 10.3389/fped.2017.00227

**Published:** 2017-10-30

**Authors:** Martha Milade Torres Nupan, Alberto Velez Van Meerbeke, Claudia Alejandra López Cabra, Paula Marcela Herrera Gomez

**Affiliations:** ^1^Neurosciences Research Group, Medicine and Health Sciences School, Universidad del Rosario, Bogota, Colombia

**Keywords:** neurofibromatosis, neurofibromatosis type 1, cognitive functioning, behavior, attention-deficit/hyperactivity disorder, autism spectrum disorder, executive functions, visuospatial functioning

## Abstract

**Aim:**

The last systematic review of research on the behavior of children with neurofibromatosis type 1 (NF1) was in 2012. Since then, several important findings have been published. Therefore, the study aim was to synthesize recent relevant work related to this issue.

**Method:**

We conducted a systematic review of the literature. Relevant articles were identified using the electronic databases PubMed, PsycINFO, and Scopus and a manual search of references lists. Thirty of 156 articles identified met the inclusion criteria. A quality evaluation of the articles was performed and the information was synthesized using a narrative approach.

**Results:**

Compared with controls, children and adolescents with NF1 present significant alterations in language, reading, visuospatial skills, motor function, executive function, attention, behavior, emotion, and social skills. The prevalence of attention-deficit/hyperactivity disorder (ADHD) is important and can affect cognition and executive function variables. A high prevalence of autistic traits and autistic spectrum disorder were reported. The benefits of using statins to treat cognitive deficits are unclear. However, children with NF1 and ADHD seem to benefit from methylphenidate treatment. The presence of hyperintensities in brain magnetic resonance imaging data seem to be related to poor cognitive performance. Analysis of these lesions could help to predict cognitive alterations in children with NF1.

**Interpretation:**

There has been important progress to evaluate cognitive characteristics of children with NF1 and to determine the physiological mechanisms of the concomitant disorders. However, discrepancies in relation to intelligence, learning disabilities, attention deficits, and treatment remain. Further investigations on this topic are recommended.

## Introduction

Neurofibromatosis type 1 (NF1) is an autosomal dominant genetic condition with a prevalence of 1 in 2,000–3,000 live births (1, 2). The main clinical manifestations include café-au-lait macules, skinfold freckling, and neurofibromas (3). The most common complications of NF1 are cognitive and behavioral deficits. Up to 80% of children with NF1 experience cognitive and behavioral difficulties involving different domains. However, the intelligence quotient (IQ) scores of these patients are within the normal range or only slightly lower compared with unaffected sibling controls. As a result, discrepancies between IQ and academic achievement are frequently observed (4). Parents often report poor performance in reading, written work, spelling, organizational skills, and mathematics. In addition, approximately 38% of affected children have attention-deficit/hyperactivity disorder (ADHD) and some studies have reported that 29% of children with NF1 have autism spectrum disorder (ASD) (5). It is important to understand these kinds of problems to identify particular needs of patients and provide individualized management of rehabilitation and educational processes.

In 2012, Lehtonen (6) published a systematic review of the literature on behavioral issues and attention disorders in patients with NF1, which identified the problems described above. Among the most important problems, these authors identified were alterations in memory, language, cognitive skills, intelligence, and academic performance and they raised many questions that remain to be clarified. The objective of this systematic review was to evaluate how much progress has been made in addressing these questions over the last 5 years. As there are many recent new research findings in this area, it is timely to update recommendations for the evaluation, follow-up, and management of NF1 patients.

## Methods

The study methods were adapted from the systematic review by Lehtonen (6).

### Eligibility Criteria

The following inclusion criteria were used: studies published from 2012 to 2016; studies with outcome variables measuring cognitive aspects, executive function, emotion, attention, or social aspects; studies that used statistical group comparisons or normative data analysis; studies using quantitative methods; studies published in peer-reviewed journals; and clinical trials. Case report studies were excluded because of potential bias. The target population was children with NF1 aged 6–17 years. Studies published in English, French, and Spanish were included.

### Search Strategy

In August 2016, an electronic databases search was done using PubMed, PsycINFO, and Scopus. For PubMed, MESH headings with OR function were used: *cognition, cognition disorders, executive function, attention, attention deficit and disruptive behavior disorders, attention deficit disorder with hyperactivity, memory, memory disorders, learning, learning disorders, behavior, motor skills, motor skills disorders, mental disorders, neurodevelopmental disorders, neurocognitive disorders, speech, speech disorders, language, language disorders, language development disorders*. The following keywords combined with the OR function were also used: *motor skills, social skills, executive function, attention, memory, cogniti$, learning, behavior, school, education, language*. Finally, the MESH heading *neurofibromatosis 1* was combined with the previous search using the AND function. Terms were adapted for use with the PyscINFO and Scopus electronic databases. The references of the included articles and the electronic sources Science Direct, Web of Science, and Springer Link were manually searched to identify additional literature.

### Study Selection and Data Extraction Process

This systematic literature review was conducted following the PRISMA reporting guidelines for systematic reviews and meta-analyses (7). Two authors (Martha Milade Torres Nupan and Claudia Alejandra López Cabra) screened all publication titles and abstracts and eliminated irrelevant articles. The full text of the remaining papers was retrieved and evaluated. Articles that did not meet the eligibility criteria were rejected. Disagreements were resolved by discussion or in consultation with the other authors (Alberto Vélez Van Meerbeke and Paula Marcela Herrera Gomez).

Two authors extracted information on population, methods, results, outcomes, level of evidence, and study quality (Martha Milade Torres Nupan and Claudia Alejandra López Cabra) (Table 1). The Joanna Briggs Institute Levels of Evidence scale was used to evaluate evidence levels. The Scottish Intercollegiate Guidelines Network methodology checklist, the Joanna Briggs Institute Critical Appraisal Checklist for Descriptive Studies, and the National Institute of Health Quality Assessment Tool for Observational Cohort and Cross-Sectional Studies were used to evaluate the quality of studies (see supporting information) (Tables 2–6). Two authors (Alberto Vélez Van Meerbeke and Paula Marcela Herrera Gomez) verified the information extracted and discrepancies were resolved by consensus (Martha Milade Torres Nupan, Claudia Alejandra López Cabra, Alberto Vélez Van Meerbeke, and Paula Marcela Herrera Gomez). All the authors evaluated the full-text articles that were retrieved. Because of the high heterogeneity of the studies, a narrative approach rather than a meta-analysis was used.

**Table 1:** Extraction table including study design, level of evidence (Joanna Briggs Institute levels of evidence), sample characteristics, objectives and results of the studies that addressed cognitive and behavior in children with neurofibromatosis type 1 (NF1).

Reference	Study design	Level of evidence	Sample	Objectives	Results
Allen et al. (27)	Case–control study	3d	23 NF1 23 Controls Age: 8–16	Identify possible relations between neurocognitive ability, facial expression recognition, and social functioning in NF1 children compared with typically developing peers	Children with NF1 had significantly lower parent- and child-rated social functioning per the Pediatric Quality of Life Inventory and greater social problems according to the Child Behavior Checklist. Children with NF1 also had significantly weaker recognition of child faces and adult faces on low intensity conditions

Aydin et al. (36)	Case–control study	3d	37 NF1 Mean age: 10.12 ± 3.82 31 Controls Mean age: 9.83 ± 3.76	Evaluate the association between the microstructural integrity of CC and neurocognitive disabilities, based on apparent diffusion coefficient and fractional anisotropy values in NF1 children compared with healthy controls	Children with NF1 showed a significantly larger total CC area than healthy controls. Apparent diffusion coefficient values obtained from the CC genu were significantly higher in NF1 children than in healthy controls. There was a negative correlation between the apparent diffusion coefficient values of the CC genu and arithmetic and digit span scores (verbal IQ and performance IQ scores), and between the fractional anisotropy values of the genu and coding scores (verbal IQ and performance IQ scores) in children with NF1

Barquero et al. (11)	Clinical trial	lc	49 NF1 + Reading deficits 17 Idiopathic reading deficit Two control groups: 14 wait list idiopathic reading deficit, 26 typically developing readers Age: 8–14	Determine the effect of remedial reading programs in children with NF1 and reading deficits	Children with NF1 and reading deficiencies responded better to the kinesthetic reading program than the one requiring visual-spatial demands. Similar distribution of reading scores in children with NF1 were found regardless of whether the Conners Parent Rating scores indicated low (T score <50), medium (T score = 50–65), or high risk (T score >65) of ADHD

Champion et al. (17)	Case–control study	3d	46 NF1 Age: 7–17 Not comparison group, normative data was used	Determine the relations between motor impairment, gait variables, and cognitive function in children with NF1	Normalized scores on the Bruininks-Oseretsky Test of Motor Proficiency, for an NF1 cohort were significantly lower than age-matched normative reference values. Compared with normative data, children with NF1 demonstrated significantly decreased performance on gait parameters. Poorer balance skills were significantly associated with reduced perceptual reasoning and working memory

Cosyns et al. (8)	Descriptive	3e	43 NF1: 14 children, age: 7.4–16; 29, adults, age: 17.9–53.5	Evaluate the articulation skills of NF1 school children and adults	Children’s phonetic inventory was incomplete for their age: realizations of the sibilants/R/and/or/a/were not totally correct. Distortions were the predominant phonetic error type and rhotacismus non vibrans were frequently observed. There were also substitution and syllable structure errors, particularly deletion of the final consonant of words. Girls tended to display more articulation errors than boys

Debrabant et al. (18)	Case–control study	3d	20 NF1 20 controls Age: 8–12	Evaluate visual-motor reaction time and its association to the impairment of fine visual-motor skills in children with NF1	Children with NF1 responded more slowly and with fewer anticipatory responses to predictive stimuli, after controlling for IQ and processing speed. Predictive reaction time performance did not differ from reaction time to unpredicted stimuli, indicating an inability to adopt rhythmic stimuli. All children with NF1 scored below the normal range (percentile 16) on the Movement Assessment Battery for Children, Second edition. Finally, the NF1 group demonstrated a significantly poorer performance on the Beery-Buktenica Developmental Test of Visual-Motor Integration copy test, showing reduced visual-motor integration and tracing outcomes (eye–hand coordination)

Galasso et al. (21)	Case–control study	3d	18 NF1 Mean age: 11.00 ± 2.87 18 ADHD Mean age: 11.17 ± 2.92 18 controls Mean age: 11.22 ± 2.80	Evaluate specific planning deficits in children with NF1 in relation to ADHD comorbidity	They found no correlation between Tower of London test scores and Conners ratings scale for parents’ scores in children with NF1. The authors concluded that planning and problem-solving deficits are not directly related to inattention level

Gilboa et al. (19, 23)	Case–control study	3d	30 NF1 Age: 8–16.6 30 controls Age: 8.4–16.3	Evaluate NF1 children performance in lower and higher processes required for intact writing; and to identify predictors of the written product’s spatial arrangement and content	Children with NF1 performed significantly poorer on higher-level processes, evaluated using the Rey Complex Figure Test for cognitive planning skills and the Hebrew version of the Wechsler Intelligence Scale for Children for verbal intelligence. Cognitive planning skills predicted the written product’s spatial arrangement and verbal intelligence scores predicted the written content level

Gilboa et al. (19, 23)	Case–control study	3d	29 NF1 Mean age: 12.3 ± 2.6 27 controls Mean age: 12.4 ± 2.5	Identify a possible relation between executive function and academic skills in children with NF1	Children with NF1 performed significantly lower on four of the BRIEF scales (initiate, working memory, plan/organize, and organization of materials) and two subtests of the BADS-C (water and key search). Significant correlations were shown between BADS-C subtest scores and ACES scale scores: children who scored higher (better performance) on the BADS-C received higher scores (better performance) from their teachers on the ACES. In addition, children who received higher scores (performed better) on the ACES received lower scores from their parents (performed better) on the BRIEF

Huijbregts et al. (37)	Case–control study	3d	15 NF1 Mean age: 12.9 ± 2.6 18 controls Mean age: 13.8 ± 3.6	Evaluate volumetric measures of cortical and subcortical brain regions in children with NF1 and its possible association with social skills, attention problems and executive dysfunction	Larger left putamen volume, larger total white matter volume, and smaller precentral gyrus gray matter density in children with NF1 were associated with more social problems (evaluated using Child Behavior Checklist parent ratings). Larger right amygdala volume in children with NF1 was associated with autistic mannerisms (evaluated using Social Responsiveness Scale parent ratings)

Isenberg et al. (12)	Case–control study	3d	55 NF1 Mean age: 9.71 ± 2.63 No control group, normative data was used	Evaluate attention skills in children with NF1 using measures of visual and sustained auditory attention, divided normative attention, selective attention, and response inhibition	Deficits in sustained visual and auditory attention, and deficits in divided attention and response inhibition were identified in Children with NF1

Lehtonen et al. (13)	Case–control study	3d	49 NF1 Mean age: 11.75 ± 3.16 19 healthy siblings Mean age: 12.58 ± 2.58 29 healthy children-community Mean age: 11 ± 2.58	Evaluate cognitive skills in children with NF1	Children with NF1 had significantly lower Full-scale IQs and lower academic achievement than their siblings. Compared with their siblings, they also had significantly poorer visuospatial processing, visual associate learning, non-verbal working memory, and executive function

Lidzba et al. (44)	Retrospective case–control study	3d	43 NF1: 16 without ADHD, 27 with ADHD (13 medicated) Age range: Tl, 6–14 years; T2, 7–16 years; mean interval, 49.09 months	Evaluate possible benefits of methylphenidate in cognitive functioning in children with NF1 and comorbid ADHD	Medicated children with NF1 improved significantly in full-scale IQ between two periods of time, this effect was not evident for the other groups. With attention measures as covariates, the effect remained marginally significant.

Lidzba et al. (25)	Retrospective Case–control study	3d	111 NF1: 36 without ADHD, 62 ADD, 13 ADHD. Age range: 6–16	Evaluation of the influence of ADHD symptoms on the intellectual profile of patients with NF1	Patients with ADHD symptoms performed significantly worse than those without ADHD symptoms on all intelligence measures (main effects for Full-scale, Verbal, and Performance IQ). Subtests typically impaired in patients with NF1 (visuospatial skills and arithmetic) were not specifically influenced by ADHD symptoms. There were no differences between ADHD subtypes

Lion-Francois etal (45)	Randomized, double blind, placebo controlled, and crossover trial	lc	39 NF1 (80 < IQ > 120) Age: 7.9–12.9	Evaluate possible benefits of methylphenidate in cognitive functioning in children with NF1 and comorbid ADHD	The Simplified Conners’ Parent Rating Scale scores decreased by 3.9 points in medicated children

Loitfelder et al. (38)	Cross-sectional study	4b	14 NF1 Mean age: 12.49 ± 2.65 30 controls Mean age: 12.30 ± 2.94	Evaluation of functional connectivity in relation to the cognitive profile of children with NF1	Associations of increased frontofrontal and functional connectivity with cognitive, social, and behavioral deficits were found. Children and adults with NF1 showed deficient activation of the low-level visual cortex and specific impairment of the magnocellular visual pathway

Michael et al. (15)	Case–control study	3d	20 NF1 20 controls Age: 7–13	Evaluation of reactivity to visual signals in children with NF1 and its alteration as a possible cause of attention instability	The NF1 group exhibited slower global responses on measures of response time and weakened resistance to interference, leading to difficulties in the ability to continuously focus on a primary task

Orraca-Castillo et al. (9)	Case–control study	3d	32 NF1 Age: 7–14 No control group, normative sample was used	Evaluate children with NF1 through neurocognitive tests dedicated to assess basic capacities which are involved in reading and mathematical achievement	Core numeric capacities do not seem to be responsible for calculation dysfluency in NF1 children. Word decoding deficits and poor number facts retrieval seem to be good predictors of dyslexia and dyscalculia, respectively. A high prevalence of developmental dyslexia was identified

Payne et al. (22)	Case–control study	3d	49 NF1 Mean age: 11 ± 2.3 35 NF1 + ADHD Mean age: 10.6 ± 2.3 30 controls Mean age: 10 ± 2.6	Evaluate if executive dysfunction is exacerbated by comorbid diagnosis of ADHD in children with NF1	Compared with typically developing children, children with NF1 with or without comorbid ADHD demonstrated significant impairment of both spatial working memory (SWM) and inhibitory control. There were no differences between the two NF1 groups in SWM or response inhibition

Payne et al. (14)	Case–control study	3d	71 NF1 Median age: 10.5 29 controls Median age: 10	Identify interrelationships between visuospatial learning and other cognitive abilities that may influence performance, such as intelligence, attention and visuospatial function in children with NF1	Children with NF1 displayed significant impairments in visuospatial learning, with reduced initial retention and poorer learning across repeated trials. Visuospatial learning was inferior in NF1 even after accounting for group differences in intelligence, sustained attention and visuospatial abilities

Payne et al. (33)	Prospective cohort study	3c	18 NF1 Age: 8–16.8 5 controls Age: 8.9–15.2	Determine the natural history of cognitive function and T2H from childhood to adulthood and to examine if the presence of discrete T2H in childhood can predict cognitive performance in adulthood	Longitudinal analyses revealed a significant increase in general cognitive function in patients with NF1 over the study period. Improvements were limited to individuals with discrete T2H in childhood. Patients without lesions in childhood exhibited a stable profile. The number of T2H decreased over time, particularly discrete lesions. Lesions located within the cerebral hemispheres and deep white matter were primarily stable, whereas those located in the basal ganglia, thalamus and brainstem tended to resolve

Piscitelli et al. (35)	Case–control study	3d	49 NF1: 32 withT2H in cerebellum, 18 without T2H. Age: 6–16.9	Evaluate the neuropsychological profile in order to establish the clinical meaning of T2H in the cerebellum of children with NF1	Patients with T2H in the cerebellum showed a lower IQ than those without. T2H-positive patients showed clinical impairment more frequently than T2H-negative patients, although the group differences were not statistically significant

Pride et al. (24)	Restrospective case–control study	3d	132 NF1 60 NF1 + ADHD 52 unaffected controls Age: 6–16	Determine if cognitive and academic functioning are affected by comorbid ADHD in patients with NF1	Children with NF1 and ADHD performed significantly worse on measures of mathematical reasoning, receptive language, sustained attention, reading, and spelling compared with children with NF1 only. Children with NF1 and ADHD were also rated more severely by parents and teachers on the BRIEF than the NF1 only group

Ribeiro et al. (16)	Case–control study	3d	17 NF1 19 controls Age: 8–17	Investigate the neural mechanisms underlying the visual deficits of children with NF1 by using visual evoked potentials and brain oscillations during visual stimulation and rest periods	Abnormal long-latency visual evoked potentials may be related to deficits in high-level processing of visual stimuli; a specific enhancement of alpha brain oscillations related to problems in attention allocation

Roy et al. (34)	Case–control study	3d	36 NF1 36 controls Age: 7–12.9 years	Compare executive functioning profile with characteristics of T2H i children with NF1	Executive dysfunction in children with NF1 was not significantly influenced by T2H presence, number, size, and location (whole brain or specific areas)

Roy et al. (26)	Case–control study	3d	30 NF1 60 controls Age: 7–12	Investigate spontaneous versus reactive cognitive flexibility in children with NF1 and their comorbidity with ADHD	NF1 children performed worse than healthy children on both spontaneous and reactive cognitive flexibility tasks, even when intelligence and basic skills were partially excluded. However, ADHD symptomatology did not adversely affect performance

Van der Vaart et al. (42)	Randomized, double-masked, placebo-controlled trial	lc	84 NF1: 43 simvastatin, 41 placebo Age: 8–16	Assess the use of simvastatin for the improvement of cognitive and behavioral deficits in children with NF1 for 12 months	Simvastatin for 12 months had no effect on full-scale intelligence, attention, and internalizing behavioral problems

Violante et al. (39)	Case–control study	3d	15 NF1 24 controls Age: 7–17	Investigate the activation pattern of high-level visual and non visual regions modulated by the different stimuli to examine possible functional consequences of low-level visual impairments	Children and adults with NF1 showed deficient activation of the low-level visual cortex, indicating that low-level visual processing deficits do not ameliorate with age. There was specific impairment of the magnocellular visual pathway in early visual processing associated with a deficient deactivation of the default mode network

Walsh et al. (30)	Retrospective, cross-sectional study	4b	66 NF1 Age: 6–12	Evaluate systematically, symptoms of autism spectrum disorder in children with NF1	Forty percent of the NF1 sample showed symptom levels that reached clinical significance on the Social Responsiveness Scale, and 14% showed levels consistent with those seen in children with autism spectrum disorder (ASD). These raised symptom levels were not explained by NF1 disease severity or externalizing and internalizing behavioral disorders. There was a statistically significant relationship between symptoms of ADHD and ASD

Wessel et al. (10)	Longitudinal cohort study	3c	124 NF1 Age: 0–8	Determine the age of presentation for specific areas of delay in children with NF1 and the time-dependent progression of these deficits	School-age children exhibited significantly more areas of delay than infants or preschool-age children. Delays in math, reading, gross motor, fine motor, and self-help development were observed more frequently in older than younger children. Analysis of 43 subjects for whom longitudinal assessments were available revealed that children often migrated between delayed and nondelayed groups in all areas except gross motor development

*ACES, Academic Competence Evaluation Scales; BADS-C, Behavioral Assessment of the Dysexecutive Syndrome in Children; BRIEF, Behavior Rating Inventory of Executive Function; CC, Corpus Callosum; ADD, Attention-Deficit Disorder; ADHD, attention-deficit/hyperactivity disorder; JLO, Judgment of Line Orientation; NF1, Neurofibromatosis type 1; T2H, T2-hyperintensities*.

**Table 2:** Quality evaluation for case–control studies using the Scottish Intercollegiate Guidelines Network (SIGN).

Reference	Sign methodology checklist: case–control studies													
	Internal validity											Overall assessment		
	1.1	1.2	1.3	1.4	1.5	1.6	1.7	1.8	1.9	1.10	1.11	2.1	2.2	2.3
Allen et al. (27)	YES	YES	NO	Cases: 96%	YES	NO	YES	NA	NA	YES	NO	(+)	NO	NO
				Controls: 100%										
Aydin et al. (36)	YES	YES	NO	Cases: 100%	YES	NO	YES	NA	NA	YES	YES	(+)	NO	NO
				Controls: 100%										
Champion et al. (17)	YES	NA	NA	Cases: 100%	NO	YES	YES	NA	NA	YES	NO	(+)	NO	NO
				Controls: normative data was used										
Debrabant et al. (18)	YES	YES	NO	Cases: 100%	YES	NO	YES	NA	NA	YES	YES	(+)	NO	NO
				Controls: 100%										
Galasso et al. (21)	YES	YES	NO	Cases: 100%	YES	NO	YES	NA	NA	YES	NO	(+)	NO	NO
				Controls: 100%										
Gilboa et al. (19, 23)	YES	YES	NO	Cases: 100%	YES	NO	YES	NA	NA	YES	NO	(+)	NO	NO
				Controls: 100%										
Gilboa et al. (19, 23)	YES	YES	NO	Cases: 100%	YES	NO	YES	NA	NA	YES	NO	(+)	NO	NO
				Controls: 100%										
Huijbregts et al. (37)	YES	CS	CS	Cases: 100%	YES	NO	YES	NA	NA	YES	YES	(+)	NO	NO
				Controls: 100%										
Isenberg et al. (12)	YES	NA	NA	Cases: 19%	NO	YES	YES	NA	NA	YES	NO	(+)	NO	NO
				Controls: normative data was used										
Lehtonen et al. (13)	YES	YES	NO	Cases: 49%	YES	YES	YES	NA	NA	YES	YES	(++)	NO	YES
				Controls: 100%										
Lidzba et al. (44)	YES	YES	NO	Cases: 100%	YES	NO	NO	NA	NA	YES	YES	(+)	NO	NO
				Controls: 100%										
Lidzba et al. (25)	YES	YES	YES	Cases: 100%	YES	YES	YES	NA	NA	YES	NO	(+)	NO	NO
				Controls:100%										
Michael et al. (15)	YES	YES	CS	Cases: 100%	NO	NO	YES	NA	NA	YES	NO	(+)	NO	NO
				Controls: 100%										
Orraca et al. (9)	YES	NA	NA	Cases: 100%	NO	YES	YES	NA	NA	YES	NO	(+)	NO	NO
				Controls: normative data was used										
Payne et al. (22)	YES	YES	NO	Cases: 90%	YES	YES	YES	NA	NA	YES	NO	(++)	NO	YES
				Controls: 100%										
Payne et al. (14)	YES	YES	NO	Cases: 95%	YES	YES	YES	NA	NA	YES	YES	(+)	NO	NO
				Controls: can’t say										
Piscitelli et al. (35)	YES	YES	YES	Cases: 100%	YES	YES	YES	NA	NA	YES	NO	(+)	NO	NO
				Controls: 100%										
Pride et al. (24)	YES	YES	NO	Cases: 100%	YES	NO	YES	NA	NA	YES	NO	(+)	NO	NO
				Controls: 100%										
Ribeiro et al. (16)	YES	YES	NO	Cases: 100%	YES	YES	YES	NA	NA	YES	NO	(+)	NO	NO
				Controls: 100%										
Roy et al. (34)	YES	NA	NA	Cases: 97%	NO	YES	YES	NA	NA	YES	NO	(+)	NO	NO
				Controls: normative data was used										
Roy et al. (26)	YES	YES	YES	Cases: 36.67%	YES	YES	YES	NA	NA	YES	NO	(+)	NO	NO
				Controls: 100%										
Violante et al. (39)	YES	YES	YES	Cases: 60%	YES	YES	YES	NA	NA	YES	NO	(+)	NO	NO
				Controls: 82%										

*NA, not applicable; CS, can’t say; (+), acceptable; (++), high quality*.

*1.1 The study addresses an appropriate and clearly focused question. Yes/no/can’t say*.

*1.2 The cases and controls are taken from comparable populations. Yes/no/can’t say*.

*1.3 The same exclusion criteria are used for both cases and controls. Yes/no/can’t say*.

1.4 What percentage of each group (cases and controls) participated in the study?

*1.5 Comparison is made between participants and non-participants to establish their similarities or differences. Yes/no/can’t say*.

*1.6 Cases are clearly defined and differentiated from controls. Yes/no/can’t say*.

*1.7 It is clearly established that controls are non-cases. Yes/no/can’t say*.

*1.8 Measures will have been taken to prevent knowledge of primary exposure influencing case ascertainment. Yes/no/can’t say/not applicable*.

*1.9 Exposure status is measured in a standard, valid and reliable way. Yes/no/can’t say*.

*1.10 The main potential confounders are identified and taken into account in the design and analysis. Yes/no/can’t say*.

*1.11 Confidence intervals are provided. Yes/no*.

*2.1 How well was the study done to minimize the risk of bias or confounding? High quality (++), acceptable (+), unacceptable (−)*.

*2.2 Taking into account clinical considerations, your evaluation of the methodology used, and the statistical power of the study, do you think there is clear evidence of an association between exposure and outcome? Yes/no/can’t say*.

*2.3 Are the results of this study directly applicable to the patient group targeted by this guideline? Yes/no*.

**Table 3:** Quality evaluation for randomized controlled trials using the Scottish Intercollegiate Guidelines Network (SIGN).

Reference	Sign methodology checklist: randomized controlled trial												
	Internal validity										Overall assessment		
	1.1	1.2	1.3	1.4	1.5	1.6	1.7	1.8	1.9	1.10	2.1	2.2	2.3
Barquero et al. (11)	YES	YES	YES	YES	YES	NO	YES	Patients with NF1 = 43%	NO	YES	(+)	NO	NO
								Patients without NF1 = 43					
Lion-Francois et al. (45)	YES	YES	YES	YES	YES	NO	YES	Placebo: 0%	YES	NA	(+)	NO	NO
								MPD: 0%					
Van der Vaart et al. (42)	YES	YES	YES	YES	YES	NO	Placebo: 4.8%	Simvastatin: 0%	YES	YES	(+)	NO	NO

*NA, not applicable; (+), acceptable; MPD, methylphenidate*.

*1.1 The study addresses an appropriate and clearly focused question. Yes/no/can’t say*.

*1.2 The assignment of subjects to treatment groups is randomized. Yes/no/can’t say*.

*1.3 An adequate concealment method is used. Yes/no/can’t say*.

*1.4 The design keeps subjects and investigators ‘blind’ about treatment allocation. Yes/no/can’t say*.

*1.5 The treatment and control groups are similar at the start of the trial. Yes/no/can’t say*.

*1.6 The only difference between groups is the treatment under investigation. Yes/no/can’t say*.

*1.7 All relevant outcomes are measured in a standard, valid and reliable way. Yes/no/can’t say*.

1.8 What percentage of the individuals or clusters recruited into each treatment arm of the study dropped out before the study was completed?

*1.9 All the subjects are analyzed in the groups to which they were randomly allocated (often referred to as intention to treat analysis). Yes/no/can’t say/not applicable*.

*1.10 Where the study is carried out at more than one site, results are comparable for all sites. Yes/no/can’t say/not applicable*.

*2.1 How well was the study done to minimize bias? Code as follows: High quality (++), acceptable (+), low quality (−), Unacceptable (−)*.

*2.2 Taking into account clinical considerations, your evaluation of the methodology used, and the statistical power of the study, are you certain that the overall effect is due to the study intervention? Yes/no*.

*2.3 Are the results of this study directly applicable to the patient group targeted by this guideline? Yes/no*.

**Table 4:** Quality evaluation for cohort studies using the Scottish Intercollegiate Guidelines Network (SIGN).

Reference	Sign methodology checklist: cohort studies																
	Internal validity														Overall assessment		
	1.1	1.2	1.3	1.4	1.5	1.6	1.7	1.8	1.9	1.10	1.11	1.12	1.13	1.14	2.1	2.2	2.3
Payne et al. (33)	YES	CS	YES	NA	Patients: 55%	NA	YES	YES	NO	YES	NO	YES	CS	NO	(+)	NO	NO
					Controls: 64.2%												
Wessel et al. (10)	YES	NA	YES	NA	CS	NA	YES	NA	NA	NA	NO	NA	YES	YES	(+)	NO	NO

*NA, not applicable; CS, can’t say; (+), acceptable*.

*1.1 The study addresses an appropriate and clearly focused question. Yes/no/can’t say*.

*1.2 The two groups being studied are selected from source populations that are comparable in all respects other than the factor under investigation*.

*1.3 The study indicates how many of the people asked to take part did so, in each of the groups being studied. Yes/no/not applicable*.

*1.4 The likelihood that some eligible subjects might have the outcome at the time of enrollment is assessed and taken into account in the analysis*.

*1.5 What percentage of individuals or clusters recruited into each arm of the study dropped out before the study was completed*.

*1.6 Comparison is made between full participants and those lost to follow up, by exposure status. Yes/no/can’t say/not applicable*.

*1.7 The outcomes are clearly defined. Yes/no/can’t say*.

*1.8 The assessment of outcome is made blind to exposure status. If the study is retrospective this may not be applicable. Yes/no/can’t say/not applicable*.

*1.9 Where blinding was not possible, there is some recognition that knowledge of exposure status could have influenced the assessment of outcome*.

*1.10 The method of assessment of exposure is reliable. Yes/no/can’t say/not applicable*.

*1.11 Evidence from other sources is used to demonstrate that the method of outcome assessment is valid and reliable. Yes/no/can’t say/not applicable*.

*1.12 Exposure level or prognostic factor is assessed more than once. Yes/no/can’t say/not applicable*.

*1.13 The main potential confounders are identified and taken into account in the design and analysis. Yes/no/can’t say*.

*1.14 Have confidence intervals been provided? Yes/no*.

*2.1 How well was the study done to minimize the risk of bias or confounding? High quality (++), acceptable (+), unacceptable (−)*.

*2.2 Taking into account clinical considerations, your evaluation of the methodology used, and the statistical power of the study, do you think there is clear evidence of an association between exposure and outcome? Yes/no/can’t say*.

*2.3 Are the results of this study directly applicable to the patient group targeted in this guideline? Yes/no*.

**Table 5:** Quality evaluation for cross sectional studies using the National Institutes of Health checklist (NIH).

Reference	Quality assessment tool for observational cohort and cross-sectional studies-NIH														
	1	2	3	4	5	6	7	8	9	10	11	12	13	14	15
Loitfelder et al. (38)	YES	YES	NR	YES	YES	NA	NA	NA	NA	NA	NO	NA	NA	NO	Fair
Walsh et al. (30)	YES	YES	NR	YES	YES	NA	NA	NA	NA	NA	YES	NA	NA	YES	Good

*NA, not applicable; NR, not reported*.

*1. Was the research question or objective in this paper clearly stated? Yes/no/not applicable/not reported*.

*2. Was the study population clearly specified and defined? Yes/no/not applicable/not reported*.

*3. Was the participation rate of eligible persons at least 50%? Yes/no/not applicable/not reported*.

*4. Were all the subjects selected or recruited from the same or similar populations (including the same time period)? Were inclusion and exclusion criteria for being in the study prespecified and applied uniformly to all participants? Yes/no/not applicable/not reported*.

*5. Was a sample size justification, power description, or variance and effect estimates provided? Yes/no/not applicable/not reported*.

*6. For the analyses in this paper, were the exposure(s) of interest measured prior to the outcome(s) being measured? Yes/no/not applicable/not reported*.

*7. Was the timeframe sufficient so that one could reasonably expect to see an association between exposure and outcome if it existed? Yes/no/not applicable/not reported*.

*8. For exposures that can vary in amount or level, did the study examine different levels of the exposure as related to the outcome (e.g., categories of exposure, or exposure measured as continuous variable)? Yes/no/not applicable/not reported*.

*9. Were the exposure measures (independent variables) clearly defined, valid, reliable, and implemented consistently across all study participants? Yes/no/not applicable/not reported*.

*10. Was the exposure(s) assessed more than once over time? Yes/no/not applicable/not reported*.

*11. Were the outcome measures (dependent variables) clearly defined, valid, reliable, and implemented consistently across all study participants? Yes/no/not applicable/not reported*.

*12. Were the outcome assessors blinded to the exposure status of participants? Yes/no/not applicable/not reported*.

*13. Was loss to follow-up after baseline 20% or less? Yes/no/not applicable/not reported*.

*14. Were key potential confounding variables measured and adjusted statistically for their impact on the relationship between exposure(s) and outcome(s)? Yes/no/not applicable/not reported*.

*15. Quality rating (good, fair, or poor)*.

**Table 6:** Quality evaluation for descriptive studies using the Joanna Briggs Institute (JBI) Critical Appraisal Checklist.

Reference	Critical appraisal checklist for descriptive studies JBI	Overall appraisal (adapted from quality assessment tool for observational cross-sectional studies NIH)								

	1	2	3	4	5	6	7	8	9	10
Cosyns et al. (8)	NA	UC	YES	YES	NA	NA	NA	YES	YES	Fair

*NA, not applicable; UC, unclear*.

*1. Was study based on a random or pseudorandom sample? Yes/no/unclear/not applicable*.

*2. Were the criteria for inclusion in the sample clearly defined? Yes/no/unclear/not applicable*.

*3. Were confounding factors identified and strategies to deal with them stated? Yes/no/unclear/not applicable*.

*4. Were outcomes assessed using objective criteria? Yes/no/unclear/not applicable*.

*5. If comparisons are being made, was there sufficient descriptions of the groups? Yes/no/unclear/not applicable*.

*6. Was follow up carried out over a sufficient time period? Yes/no/unclear/not applicable*.

*7. Were the outcomes of people who withdrew described and included in the analysis? Yes/no/unclear/not applicable*.

*8. Were outcomes measured in a reliable way? Yes/no/unclear/not applicable*.

*9. Was appropriate statistical analysis used? Yes/no/unclear/not applicable*.

*10. Quality rating (good, fair, or poor)*.

## Results

**Figure 1:**
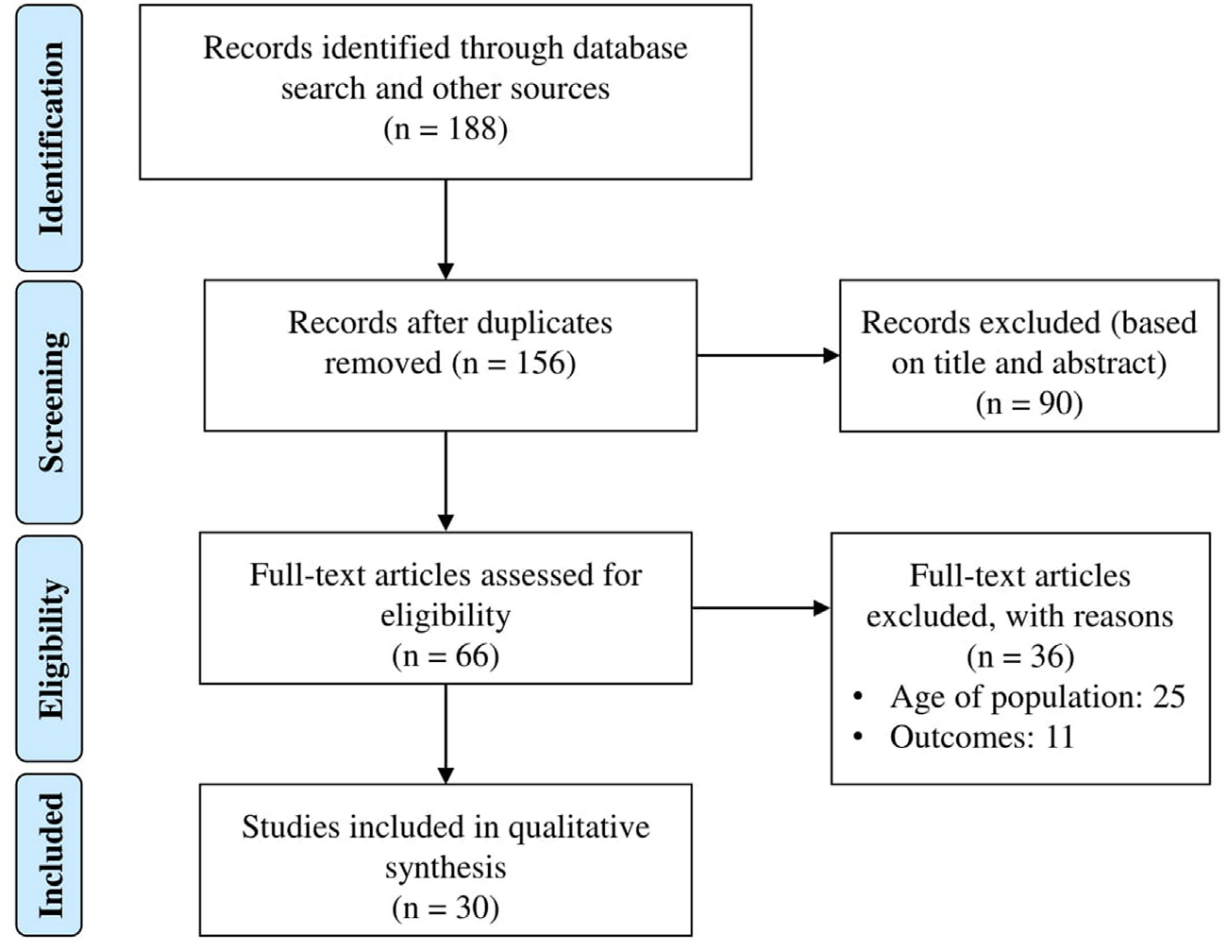
Systematic review flow diagram.

The database search identified 158 papers (Figure 1). Thirty additional articles were identified from other sources. After remove duplicates, the titles and abstracts of 156 articles were screened. During the first selection process, 90 studies were excluded. Sixty-six full-text articles were assessed for eligibility; of these, 36 were excluded because of different age groups and outcomes. Thirty articles that fulfilled the inclusion criteria were taken into account for the qualitative analysis.

### Language, Reading, and Mathematics

Several studies have shown deficits in language, reading, and reading comprehension in children with NF1. However, evaluations of these abilities differ, making it difficult to identify problems in specific aspects of each ability. Revising current literature, there is a lack of information (in both English and other languages) on speech production in children with NF1 with only one article related to this topic in the last 5 years. In this article, Cosyns (8) studied phonetic articulation in 29 Flemish adults and 14 schoolchildren (>7 years) with NF1 using a standardized speech test for Flemish single speech sounds. They found that the children’s phonetic inventory was incomplete for their age: realizations of the sibilants/R/and/or/a/were not correct. Distortions were the predominant phonetic error type and rhotacismus non-vibrant were frequently observed. There were also substitution and syllable structure errors, particularly deletion of the final consonant of words. Girls tended to display more articulation errors than boys did. More studies in different languages are needed to obtain a deeper understanding of this aspect of speech production.

Orraca-Castillo (9) evaluated mathematics and reading skills in 32 NF1 children using the Mathematics Attainment Test and the Reading and Comprehension Attainment Test. They also used two computer-based tests to measure simple reaction times from the Basic Numerical Battery and the word and pseudoword-reading task from the *Batería de Trastornos de la Lectura*. Lexical/phonological skills and mental arithmetic were significant predictors of individual differences in reading achievement and math. The sample showed no deficits in core numeric capacities, suggesting that these are not responsible for calculation dysfluency. Word decoding deficits and poor number facts retrieval seem to be good predictors of dyslexia and dyscalculia, respectively. Interestingly, Orraca-Castillo found no gender differences and identified a 50% prevalence of developmental dyslexia in their sample, a cooccurrence almost three times higher than dyscalculia (18.8%). In concordance with these results, Wessel (10) using a sample of 124 NF1 participants between 0 and 8 years, showed that school-age children with NF1 exhibited significantly more delays in reading and math than infants or preschool-age children with NF1, using the Parents’ Evaluation of Developmental Status as a tool of assessment. These findings are in line with previous reports that NF1 patients show a high prevalence of learning disorders not related to IQ achievement discrepancies.

Barquero et al. (11) conducted the first evaluation of an intervention for reading deficiencies in 49 children with NF1. Some children received remedial reading instruction from a kinesthetic reading program and others used a program that required greater visuospatial demands. Children with NF1 and reading deficiencies responded better to the kinesthetic reading program. However, the baseline mean reading score of the group using the program that required greater visuospatial demands was significantly higher than that of the group receiving the kinesthetic treatment.

### Visuospatial Functioning

Visuospatial deficits are considered a “hallmark” phenotypic characteristic of patients with NF1. The Judgment of Line Orientation (JLO) is one of the main tests used to assess these deficits because it is more consistent than other tools. Using the JLO test, Isenberg et al. (12) and Lehtonen et al. (13) compared the visuospatial processing of children with NF1 to their siblings or healthy children. Lehtonen et al. (13) showed that children with NF1 performed significantly poorer than their siblings. Isenberg et al. (12) found that the visuospatial processing of children with NF1 was below the age-based normative data, although the SD range was relatively large, indicating a high variability within the study.

Lehtonen et al. (13) and Payne et al. (14) compared visuospatial learning in children with NF1 with that of their siblings using the paired associate learning task from the Cambridge Automated Neuropsychological Test Assessment Battery. Lehtonen (13) found that NF1 children made more mistakes than their siblings on the total number of errors, but this difference was not statistically significant. Payne et al. (14) found that children with NF1 showed important deficits in visuospatial learning, with bad initial memorization and poorer learning in repeated trials, even after explaining group differences in intelligence, sustained attention, and visuospatial skills. Defective visuospatial learning was identified as a major phenotypic trait in children with NF1.

Michael et al. (15) compared reactivity to visual signals in 20 children with NF1 against 20 controls. They used a visual discrimination task with targets and distractors taken from the Living English Structure for Schools pictures. The NF1 group exhibited slower global responses on measures of response time and weakened resistance to interference, leading to difficulties in the ability to continuously focus on a primary task. The authors suggest that NF1 is characterized by over-reactivity to, and longer inspection of, visual signals occurring outside the current focus of attention and that this might be partially responsible for instability in attentional focus and lower interference resistance.

Electrophysiological tools have also been used to study the mechanisms of visuospatial deficits. Ribeiro et al. (16) studied the neural mechanisms underlying visual deficits in children with NF1. He found abnormal long-latency visual evoked potentials that may be related to deficits in process visual stimuli; in addition, they identified an increase of alpha brain oscillations probably due to problems in attention allocation.

### Visuomotor Function, Motor Control, and Coordination

Children with NF1 show motor impairments, such as alterations in simple and complex motor tasks and deficits in visuomotor function. However, the associations between these characteristics remain unclear. Regarding motor performance, Champion (17) found that normalized scores on the Bruininks–Oseretsky Test of Motor Proficiency, Second edition, for an NF1 cohort of 46 participants, were lower than the normative reference-age valuescompared with normative data; children with NF1 demonstrated a significant decrease in speed, cadence, stride length, individual support and support base. A longer step time and double support were also observed. This study also showed that poor balance skills are associated with difficulties in perceptual reasoning and working memory. The decrease in speed in running and agility were related to worse spatial working memory (SWM), working memory, and perceptual reasoning. The authors found a significant association between walking gait width with SWM decrease and the same between short steps with a poorer SWM strategy.

Debrabant et al. (18) evaluated visual-motor reaction time and its association with the grade of impaired fine visual-motor skills and compared these parameters in 20 NF1 children against 20 controls. After controlling for IQ and processing speed, it was shown that the response of children with NF1 was slower with fewer anticipatory responses to predictive stimuli. Also, the predictive performance of the reaction time did not differ from reaction time to unexpected stimuli, indicating an inability to take rhythmic stimuli. Furthermore, all children with NF1 had abnormal scores (<16th percentile) on the battery of the assessment of movement for children, second edition. Finally, NF1 group demonstrated a significantly poor performance in the Beery-Buktenica developmental test of visual-motor integration, which is interpreted as a reduction of visual-motor integration and tracing outcomes (hand–eye coordination).

Wessel et al. (10) studied developmental delays in 124 children with NF1 using the Parents’ Evaluation of Developmental Status. In relation to motor control, they found delays in the development of gross motor, fine motor, and self-help. This was mainly observed in school-age children when compared to preschool children or infants. Longitudinal evaluations of 43 subjects revealed that children often exchanged from the delayed group to the not delayed one, in all areas except gross motor development.

### Intelligence and Thinking

Children with NF1 have a slightly lower performance on intelligence tests than healthy children, and generally show mean or below-average scores on IQ. Champion et al. (17) and Lehtonen et al. (13) evaluated intellectual ability in NF1 children and comparison groups using the Wechsler Intelligence Scale for Children. Lehtonen et al. (13) found that children with NF1 had a mean full-scale IQ of 91, whereas the sibling comparison group had a mean IQ score of 99 (a statistically significant difference). NF1 children also showed lower academic achievement. Champion et al. (17) found that children with NF1 had a mean IQ of 86 and had scores significantly below normative data on all IQ variables.

Gilboa et al. (19) have shown that children with NF1 performed significantly poorer on higher-level processes, evaluated using the Rey Complex Figure Test for cognitive planning skills and the Hebrew version of the Wechsler Intelligence Scale for Children for verbal intelligence. Cognitive planning skills predicted the spatial arrangement of the written product and verbal intelligence scores predicted the level of written content. In contrast, participants in the Isenberg study (12) showed an average intellectual capacity in the Peabody Picture Vocabulary Test.

### Executive Function

The executive functions comprise all the processes that help to monitor and regulate cognitive processes during complex tasks and include planning, self-regulation, behavior organization, cognitive flexibility, working memory, error detection and correction, inhibition, sustained attention, and resistance to interference. As different measures have been used to evaluate executive functions, it is hard to make comparative validations of distinct scales and evidence remains inconclusive (6, 20). Comorbidity with ADHD is also thought to affect executive function assessment, although this correlation is not well understood.

Lehtonen et al. (13) and Champion et al. (17) evaluated executive function in children with NF1 against comparison groups using the Behavior Rating Inventory of Executive Function (BRIEF). Lehtonen et al. (13) reported poorer performance of NF1 children, who scored 1.5 SDs or more, below the normative mean. Champion et al. (17) examined parent ratings of the functional executive skills of children and adolescents with NF1. Assessments were made using the Behavioral Regulation Index, the Metacognition Index, and the Global Executive Composite. They found that participants showed significantly more behavioral executive difficulties than expected from the normative data.

Galasso et al. (21) studied individual features of executive function as planning aspects and their association with attention. They found no correlation between Tower of London test scores and Conners ratings scale for parents’ scores in children with NF1. The authors concluded that planning and problem-solving deficits are not directly related to inattention level. In concordance with these results, Payne et al. (22) showed that executive dysfunction occurred with the same severity in children with NF1, whether or not they had a comorbid diagnosis of ADHD (10).

Gilboa et al. (23) evaluated executive function in 29 children with NF1 and 27 typically developing controls using the Behavioral Assessment of the Dysexecutive Syndrome in Children (BADS-C) and the BRIEF parent questionnaires. The Academic Competence Evaluation Scales (ACES) were used to evaluate academic success. Children with NF1 performed significantly lower on four of the BRIEF scales (initiate, working memory, plan/organize, and organization of materials) and two subtests of the BADS-C (water and key search). Academic skills scores of children with NF1 and typically developing controls fluctuated significantly on the ACES teacher questionnaire variables. Significant correlations were shown between BADS-C subtest scores and ACES scale scores: children who scored higher (better performance) on the BADS-C received higher scores (better performance) from their teachers on the ACES. In addition, children who performed better in the ACES also obtained better performance in the BRIEF questionnaire conducted by the parents.

### Attention

Attention deficits have been linked to the NF1 phenotype and show an incidence of 40–50% in NF1 patients (6). The presence of ADHD in children with NF1 is associated with poorer performance in cognitive function, learning, social skills, and academic achievement. However, the impact of ADHD on these abilities, and its association with executive dysfunction, require further study.

Isenberg et al. (12) compared the performance of 55 children with NF1 with normative data for specific measures of attention. They compared mean scores for NF1 patients on each of the dependent Test of Everyday Attention for Children subtest variables with the population means. Sustained auditory attention and divided auditory attention evaluated using the score and score DT, respectively, differed significantly. The Conners Continuous Performance Test—2nd Edition was also used to evaluate attention. NF1 children’s scores showed greater omissions and commissions compared with the general population mean. No significant differences were found for hit reaction time, block change, or hit standard error block change. Based on parental responses on the Conners 3rd Edition—Parent scale, a large portion (23 of the 55 participants) of the sample met the ADHD criteria of the Diagnostic and Statistical Manual of Mental Disorders—4th Edition, Text Revision, but children with NF1 and ADHD did not differ significantly from children without ADHD on attention measures.

Pride et al. (24) showed that NF1 and ADHD children had a poorer performance in measures of mathematical reasoning, receptive language, sustained attention, reading, and spelling compared with children with NF1 only. In addition, he found that visuospatial planning and skills (assessed using the Tower of London and JLO tests, respectively) predicted academic performance in children with NF1 only. On the BRIEF test, parents and teachers also rated children with NF1 and ADHD more severely than in the NF1 only group. ADHD symptom severity (Conners ADHD/DSM-IV Scales—inattentive index) and a measure of attentional control (Test of Everyday Attention for Children—creature counting) were the best tests to predict academic performance in both groups. In children with NF1 and ADHD, BRIEF scores (Global Executive parent version) was more effective in predicting academic performance. Supporting these findings, Lidzba et al. (25) showed that patients with ADHD symptoms had significantly worse outcomes than those without ADHD symptoms in main effects full-scale, verbal, and performance-related IQ, with no differences between ADHD subtypes.

However, usually ADHD symptoms were not influenced by poor outcomes of the subtest in patients with NF1 (visuospatial skills and arithmetic). Although further cognitive impairment could be predicted in children with NF1 and concomitant ADHD, some studies have shown no difference in specific skills between NF1 children with ADHD and children with NF1 only. Moreover, Barquero et al. (11) found that scores for ADHD symptoms according to Conners Parent Rating were similar in the four reading groups assessed (NF1 and reading impairment, idiopathic reading deficit, reading deficit reading group, and typical development). In addition, Payne et al. (22) studied SWM and inhibitory control in NF1 children with and without comorbid ADHD. Compared with typically, developing children, both groups demonstrated significant impairments in these aspects and there were no between-group differences. Finally, Roy et al. (26) reported that NF1 children had worse outcomes than control children on tasks of spontaneous and reactive cognitive flexibility, even when intelligence and basic skills were partially excluded. However, ADHD symptoms did not negatively impact performance on tests.

### Emotionality, Behavior Problems, and Social Competence

Parent evaluations of NF1 children are an appropriate indicator of behavioral, social, and emotional performance. In relation to theses aspects, Allen et al. (27) reported that scores on the Pediatric Quality of Life Inventory indicated that children with NF1 had significantly lower parent- and child-rated social functioning and lower overall parent-rated emotional functioning. Scores on the Child Behavior Checklist showed that these children also displayed more internalizing and externalizing problems and greater social problems than typically developing peers. A trend was noted for parents’ ratings on social problems and inattention in NF1 children, indicating a positive association between inattention and social problems. Participants also completed a measure of facial expression recognition, the Diagnostic Analysis of Nonverbal Accuracy–Revised. Children with NF1 recognized less the faces of children and adults in low intensity conditions.

### Autism

Neurofibromatosis type 1 children show poorer social skills and social competence and a high percentage of individuals with NF1 have social deficits. Attention deficits have an important effect on poor social outcomes (5, 28–30). However, the link between attention and social competence deficits in children with NF1 remains unclear. Several articles on ASD in patients with NF1 have been written in recent years. Here, we report only the findings of Walsh et al. (30), as this article was the only one that met the inclusion criteria.

Walsh et al. (30) evaluated ASD symptomatology in 66 children with NF1. Forty percent of the children with NF1 presented symptoms at levels with clinical significance on the Social Responsiveness Scale, and 14% of this group showed symptoms at the levels observed in children with ASD. The increased of the symptom levels was not explained byNF1 severity or externalizing and internalizing behavioral. The relationship between symptoms of ADHD and ASD was statistically significant. There were also interesting relationships between ADHD and deficits in the social domain of conscience and motivation.

### Neuroimaging

One of the findings described in magnetic resonance imaging (MRI) are circumscribed areas of hyperintensity in T2-weighted imaging (T2H). These lesions seem to represent areas of demyelination or increased fluid within the myelin sheath and have been associated with cognitive impairment. However, these lesions tend to disappear over time and the relation between lesion presence, lesion quantity, and cognitive impairment is unclear (31, 32). Payne et al. (33) followed the course of T2H and cognitive function in 18 children with NF1 for 18 years. They found a significant improvement in general cognitive function in NF1 patients over the follow-up. The increase in cognitive function was restricted to participants with discrete T2H in childhood. Patients without lesions in childhood exhibited unchanged performance in cognitive function. The amount of T2H, mainly in the case of discrete lesions, decreased over time. Lesions in the cerebral hemispheres and deep white matter remained stable. The lesions located in the basal ganglia, thalamus, and brainstem resolved in many cases. The authors suggest that the presence of T2H can predict cognitive performance in childhood but not in adulthood.

Two articles reported additional interesting results related to T2H. Roy et al. (34) found that the presence, number, size, or location of T2H lesions did not affect executive dysfunction in NF1 children. On the other hand, Piscitelli et al. (35) found that patients with T2H in the cerebellum showed a lower IQ than patients without T2H in that brain location, possibly because of impaired visuospatial ability and language. Clinical impairment was more frequent in patients with T2H in comparison with those without these lesions, although the group differences were not statistically significant.

Intracranial manifestations of NF1 are also of interest, but their pathogenesis and effects on neurocognitive function have not been clearly described. Regarding this aspect, Aydin et al. (36) studied the relationship between corpus callosum (CC) characteristics and neurocognitive impairment in children with NF1 using diffusion tensor imaging features. Children with NF1 showed a significantly larger total CC area than healthy controls. The values of apparent diffusion coefficient for the CC genu were significantly higher in NF1 participants in comparison with healthy controls. The correlations between the values of apparent diffusion coefficient for the CC genu and the digit and arithmetic span scores (verbal IQ and performance IQ scores) was negative, and between the fractional anisotropy values of the genu and coding scores (verbal IQ and performance IQ scores) in NF1 participants were negative. Huijbregts et al. (37) used MRI scanning to study cerebral volumetric abnormalities in children with NF1. They found that larger left putamen volume, larger total white matter volume, and smaller precentral gyrus gray matter density in children with NF1 were associated with a higher rate of social problems (evaluated using Child Behavior Checklist parent ratings). Larger right amygdala volume in children with NF1 was associated with autistic mannerisms (evaluated using Social Responsiveness Scale parent ratings).

Resting-state functional imaging has been used to assess the integrity of brain functional connectivity networks, as abnormal function is related to neurocognitive deficits. Using resting-state functional scanning, Loitfelder et al. (38) studied functional connectivity. In patients with NF1, there were associations between the frontal and temporo-frontal functional connectivity of the ventral anterior cingulate ventral cortex, bilateral amygdala, bilateral orbitofrontal cortex, and posterior cingulate cortex with cognitive, social, and behavioral deficit. Violante et al. (39) using functional MRI to study early visual cortical pathways found that children and adults with NF1 showed poor activation of the low-level visual cortex. This may be interpreted, as that low-level visual processing disorder do not improve over time. There was a specific impairment of the magnocellular visual pathway in early visual processing related to poor default network deactivation. This evidence may help to understand the neural substrate for higher order (specifically, visuospatial and attentional) cognitive deficits present in NF1.

### Pharmacological Interventions

Statins such as lovastatin, which inhibit HMG-CoA reductase, acted on synaptic plasticity and improved learning and attention deficits in a mouse model of NF1 (40). Payne et al. (41) evaluated the effects of lovastatin in children with NF1 who previously demonstrated impairments of visuospatial learning and attention. Lovastatin had no significant effect on primary outcomes (visuospatial learning and sustained attention) after 16 weeks of treatment. The medication was well tolerated and did not increase adverse events compared with a placebo. Van der Vaart et al. (42) studied the results of simvastatin use on cognitive deficits and behavioral disorders in patients with NF1. Twelve months of treatment had no effect on intelligence, attention, and internalizing of behavioral problems. However, more studies in humans are needed to evaluate these effects.

Methylphenidate (MPD), a psychostimulant frequently used for ADHD due to its action on dopamine and the noradrenergic system (43), may have beneficial effects in patients with NF1 (43). Lidzba et al. (44) studied the effects of MPD on cognition in children with NF1 and ADHD. They showed that the full-scale IQ of medicated children with NF1 improved between two periods, an effect not evident in other groups. When attention measures were used as covariates, the effect remained marginally significant. Lion-Francois et al. (45) also studied the effects of MPD in NF1 children with ADHD-like symptoms. They found that Simplified Conners’ Parent Rating Scale scores decreased by 3.9 points in medicated children. In contrast to these results, Isenberg et al. (12) found that parental responses on the Conners 3rd Edition—Parent scale indicated that children on treatment with stimulant medication were more inattentive and oppositional than children not on medication.

## Discussion

### Cognitive Skills and Intelligence

Neurofibromatosis type 1 children show to some extent, lower performance than their typically developing peers on intelligence tests do, with 90% IQ scores between low and average range. However, up to 40% of NF1 children present learning disabilities. Difficulties with academic performance are also common. Cognitive impairment in these children may be related to social difficulties, attention deficit, behavioral and emotional problems (4, 6, 46, 47). Disagreement persists about the precise IQ profile of children with NF1. Most articles included in this review indicated a slightly below average performance on all IQ variables. However, significantly lower full-scale IQ has also been reported (17). In particular, articles mentioned poor performance in cognitive planning skills, verbal intelligence, and working and non-working memory. Klein-Tasman et al. (48) found similar results for young children (3–6 years of age) with NF1, almost half of who showed cognitive vulnerabilities.

In relation to language, deficits in phonological skills and receptive language have been described in children with NF1. There are insufficient studies to confirm the consistency of these findings and the nature of deficits in specific areas of linguistic development is still unclear. Recent findings support earlier results showing significant alterations in language, reading, and spelling in children with NF1 (4, 6, 47). School-age children exhibit significantly more delays in reading and math than preschool-age children (10). Competence related to lexical/phonological strategies and mental arithmetic seems to be a significant predictor of differences among individuals in the acquisition of reading skills and math (9). Orraca-Castillo et al. (9) replicated the findings of Watt et al. (49) and showed a high prevalence of dyslexia (greater than 50%) in this population.

Speech production in children with NF1 has not been widely studied. We reviewed a study by Cosyns et al. (8) on speech quality in Flemish adults and children. Their findings supported previous work by Alivuotila et al. (50) who showed that difficulties experienced by children with NF1 in producing speech sounds do not seem to be language dependent, but may be a particular trait of this condition. Brei et al. (51) reported that young children of 4–6 years had an increased risk of language difficulties. Although deficits in the auditory temporal processing have demonstrated an association with cognitive impairment and language deficits (52), these aspects have not been well studied in recent years.

In relation to visuospatial function in children with NF1 (53), recent evaluations using the JLO test showed poor performance compared with healthy siblings (12, 13). Evaluations of visuospatial learning using paired associate learning tests from the Cambridge Automated Neuropsychological Test Assessment Battery also indicate significant impairment in children with NF1 (13, 14). Research on the neural mechanisms of visuospatial deficits indicates that abnormal long-latency visual evoked potentials and specific enhancement of alpha brain oscillations are related to problems in attention allocation (16). Slower global responses and weakened resistance to interference in reactivity to visual signals have also been reported (15).

Recent studies on motor function indicate deficits in motor skills associated with poorer reasoning and working memory indexes (17), supporting previous results (54). Reduced visual-motor integration and poor tracing outcomes evaluated using the Beery–Buktenica Developmental Test of Visual-Motor Integration copy test have also been reported (18). Wessel et al. (10) found delays in gross and fine motor responses. Interestingly, using longitudinal assessments, they showed that children changed frequently between delayed and non-delayed groups in most areas excluding gross motor development. These patients also showed an inability to adopt rhythmic stimuli. A study by Iannuzzi et al. (55) (not included because of age criteria) suggested that fine motor skills impairment in young NF1 children may result from a comorbidity between NF1 and language disorders. More studies are needed to understand this possible correlation.

### Executive Function and Attention

Evaluations using the BRIEF tests have shown poor executive function in children with NF1. Reports also indicate greater executive behavioral difficulties than expected (13, 17), but no correlations between the planning aspects of executive function and attention. Impairment of executive function seems to be independent of attention (21, 22). Lehtonen et al. (6) had suggested the importance of studying academic achievement in relation to executive function. Gilboa et al. (23) examined these abilities and found a significant correlation between poor executive function and academic difficulties, suggesting that academic performance depends on several cognitive and behavioral domains (4). Studies of adolescents have shown similar significant correlations between executive dysfunction and academic achievement (56). Executive function deficits have been reported as a core feature of NF1, not a secondary effect derived from other variables (57).

Some research indicates that compared with children with NF1 only, children with NF1 and ADHD have a poorer performance on all cognitive attentional measures and on executive function (24, 25). In contrast, several studies indicate the presence of attention deficits in children with NF1 that do not seem to affect the performance of specific skills compared with children with NF1 only. Reading (11), spontaneous and reactive cognitive flexibility tasks (26), SWM and inhibitory control (22) were not adversely affected by ADHD symptomatology. The association between attention deficits and motor skills has not been well studied. A study by Casnar et al. (58) (not included here because of age criteria) found no correlation between fine motor skills and parental reports of attention in younger NF1 patients. Further studies in this area are needed.

### Emotionality, Behavior Problems, and Social Competence

Research findings on emotion, behavior, and social competence have been replicated by Allen et al. (27). Generally, children with NF1 show lower emotional and social functioning and more internalizing and externalizing problems. Facial expression recognition is weaker in low-intensity conditions and inattention is related to greater social problems in children with NF1. Importantly, it has been demonstrated that NF1 patients also experience a higher degree of loneliness than siblings (59) and lower participation in activities that require development of specific skills, including physical activity (60), more anxiety symptoms (61, 62), and more sleep disturbances (63). In particular, children with NF1 and plexiform neurofibromas have positive scores in the significant risk/clinical ranges on several parent and teacher subscales of the Behavior Assessment System for Children—Second Edition, including somatization, attention problems, depression, and withdrawal (64). These problems should be considered, as they contribute to poorer cognitive performance, social interaction, and emotional function. Children with NF1 could benefit from interventions focused on social skills and emotional support.

There are several recent articles on ASD in patients with NF1. Here, we report only the results of Walsh et al. (30), as this was the only article that met the age criteria. Generally, studies suggest a higher prevalence of autistic traits and ASD among patients with NF1 (5, 30, 65, 66) and the cooccurrence of ASD symptoms with ADHD symptoms (30, 66). Some researchers have tried to characterize the phenotypic profile of ASD in patients with NF1. Findings indicate that children with NF1 who have ASD symptoms tend to show more social impairment than restrictive/repetitive behaviors (28, 29). Regarding screening tools for ASD symptoms in children with NF1, no significant differences in screen-positive rates between children with NF1 and controls have been reported (67).

### Neuroimaging

Previous studies indicate that children with NF1 and T2H have lower IQ scores and poorer visuospatial and fine motor skills (68, 69). Severe impairment is particularly associated with lesions in the thalamus (70, 71). Recent studies of T2H mention findings in relation to presence, location, and cognitive impairment. NF1 patients with T2H in the cerebellum show lower IQs than those without (35). However, executive function is not influenced by T2H in NF1 children (34). One study not included here because of age criteria reported no correlation between the presence of T2H in MRI of any brain region and ratings on the social/emotional parameters of the Strengths and Difficulties Questionnaire in children with NF1 (72).

Evidence of improvement in cognitive ability over time in NF1 patients with T2H remains controversial. In 2003, Hyman et al. (31) reported a significant decrease of T2H in terms of number, size, and intensity over an 8-year period in NF1 children that was not related to changes of cognitive ability in adulthood. In contrast, another study found improvements in cognitive function over an 18-year period in children with discrete T2H (33). The disparity in these results may be related to the time of follow-up and the characteristics of T2H. More studies are needed to clarify this association.

Regarding intracranial manifestations of NF1, positive associations have been found between volume of specific brain structures and social problems (assessed using Child Behavior Checklist parent ratings and Social Responsiveness Scale ratings of autistic mannerisms) (36, 37). Based on previous reports of CC enlargement in children with NF1, Aydin et al. (36) examined the association between CC integrity and cognitive disabilities in children with NF1 and healthy controls. Diffusion tensor imaging showed no association between larger CC area and IQ scores but (compared with controls) there was a negative correlation between higher apparent diffusion coefficient and fractional anisotropy values of the genu and poorer arithmetic and verbal performances. Loitfelder et al. (38) used resting-state functional imaging and found associations of increased frontofrontal and functional connectivity with impairment at social, cognitive, and behavioral levels. Children and adults with NF1 showed weak activation of the low-level visual cortex and specific impairment of the magnocellular visual pathway (39).

### Pharmacological Interventions

Although lovastatin reverses learning deficits in a mouse model of NF1 (40), the benefits of this medication have not been clearly demonstrated in humans. The studies we reviewed of statins did not show positive effects on children with NF1. Lovastatin did not have a significant effect on visuospatial learning and sustained attention in NF1 children and simvastatin did not have on attention, full-scale intelligence, and internalizing behavioral problems. A recent study of lovastatin and neurobehavioral function (not included here because of age criteria) showed beneficial effects of this medication on some functions related to learning and memory and on internalizing symptoms in patients aged 10–50 years (73). Further studies are needed to identify possible benefits in humans.

Methylphenidate has shown benefits by decreasing ADHD symptomatology and has a good risk-benefit profile (43, 74, 75). Therefore, the effects of MPD on cognition in children with NF1 and ADHD have been evaluated. The full-scale IQ scores of medicated children with NF1 improved significantly and MPD also decreased simplified Conners scores (45). In contrast, one study reported that children taking stimulant medication were more inattentive and oppositional than children not on medication according to Conners 3rd Edition—Parent ratings (12). More studies are needed to corroborate these effects.

## Methodological Issues

Most of the studies recruited samples from medical centers, clinics, schools, and clinical trial websites. In some studies, the method of recruitment was not completely clear and information about the number of participants at the start and end of the study was lacking. Siblings, community, and normative data were used as comparison groups. It is important to point out that it is not always easy to interpret results and establish correlations owing to differences between samples and comparison groups (6).

Several studies included in this review did not control for IQ differences in analyses [only three studies controlled for IQ (13, 18, 27)]. Whether IQ should be controlled as a covariate remains controversial. Dennis et al. (76) found that several anomalous results in neurocognitive function studies have been caused by using IQ as a covariate. It has also been suggested that many differences disappear when IQ is controlled for; however, there are no recommendations of when IQ control should be used (6). Another concern is the adequacy of the tools to measure cognitive and behavioral functions. These constructs are complex and aspects of these functions (particularly executive function) frequently overlap. Evaluating the theoretical background of these constructs remains important (6). We did not include ongoing and unpublished studies and we did not identify any non-English language articles that met the inclusion criteria.

## Future Directions

Future research on NF1 should evaluate those aspects of the disorder for which findings remain inconclusive. Analysis of the interaction between cognitive and social processes may generate important information that could guide social interventions (27). In addition, reading interventions could be conducted with larger samples and in combination with pharmaceutical interventions, such as lovastatin (11, 73) and MPD, which have shown some benefits for cognitive function (45). More studies on motor skills are needed to clarify the interdependency between these skills and neurocognitive performance (17), attention deficits (58), and language (55). Systematic investigation of metacognitive constructs to obtain more conclusive results remains an important issue (6). Given the suggested relation between deficits in executive function and poor academic performance in NF1 children, the study of interventions to improve executive skills could make an important contribution to the management and follow-up of these patients (56). Finally, investigations using neuroimaging techniques could provide a better understanding of the mechanisms involved in cognitive and social functioning in children with NF1 (35, 37).

## Author Contributions

AM and PG provided conceptual guidance on the manuscript. MN, AM, PG, and CC wrote the manuscript.

## Conflict of Interest Statement

The authors declared no potential conflicts of interest in relation to the research, authorship, and publication of this article.

## Abbreviations

ACES, academic competence evaluation scales; ADHD, attention-deficit/hyperactivity disorder; ASD, autism spectrum disorder; BADS-C, behavioral assessment of the dysexecutive syndrome in children; BRIEF, behavior rating inventory of executive function; CC, corpus callosum; JLO, judgment of line orientation; MPD, methylphenidate; MRI, magnetic resonance imaging; NF1, neurofibromatosis type 1; SWM, spatial working memory; T2H, T2-hyperintensities.

## References

[1] Evans DG Howard E Giblin C Clancy T Spencer H Huson SM Birth incidence and prevalence of tumor-prone syndromes: estimates from a UK family genetic register service. Am J Med Genet A (2010) 152A(2):327–32. 10.1002/ajmg.a.33139 20082463

[2] Lammert M Friedman JM Kluwe L Mautner VF. Prevalence of neurofibromatosis 1 in German children at elementary school enrollment. Arch Dermatol (2005) 141(1):71–4. 10.1001/archderm.141.1.71 15655144

[3] DeBella K Szudek J Friedman JM. Use of the national institutes of health criteria for diagnosis of neurofibromatosis 1 in children. Pediatrics (2000) 105(3 Pt 1):608–14. 10.1542/peds.105.3.608 10699117

[4] Schwetye KE Gutmann DH. Cognitive and behavioral problems in children with neurofibromatosis type 1: challenges and future directions. Expert Rev Neurother (2014) 14(10):1139–52. 10.1586/14737175.2014.953931 25161109

[5] Garg S Green J Leadbitter K Emsley R Lehtonen A Evans DG Neurofibromatosis type 1 and autism spectrum disorder. Pediatrics (2013) 132(6):e1642–8. 10.1542/peds.2013-1868 24190681

[6] Lehtonen A Howie E Trump D Huson SM. Behaviour in children with neurofibromatosis type 1: cognition, executive function, attention, emotion, and social competence. Dev Med Child Neurol (2013) 55(2):111–25. 10.1111/j.1469-8749.2012.04399.x 22934576

[7] Moher D Liberati A Tetzlaff J Altman DG Preferred reporting items for systematic reviews and meta-analyses: the PRISMA statement. PLoS Med (2009) 6(7):e1000097 10.1016/j.ijsu.2010.02.007 19621072 PMC2707599

[8] Cosyns M Mortier G Janssens S Bogaert F D’Hondt S Van Borsel J. Articulation in schoolchildren and adults with neurofibromatosis type 1. J Commun Disord (2012) 45(2):111–20. 10.1016/j.jcomdis.2011.11.002 22192635

[9] Orraca-Castillo M Estevez-Perez N Reigosa-Crespo V Neurocognitive profiles of learning disabled children with neurofibromatosis type1. Front Hum Neurosci (2014) 8:386 10.3389/fnhum.2014.00386 24936179 PMC4048011

[10] Wessel LE Gao F Gutmann DH Dunn CM. Longitudinal analysis of developmental delays in children with neurofibromatosis type 1. J Child Neurol (2013) 28(12):1689–93. 10.1177/0883073812462885 23112244

[11] Barquero LA Sefcik AM Cutting LE Rimrodt SL. Teaching reading to children with neurofibromatosis type 1: a clinical trial with random assignment to different approaches. Dev Med Child Neurol (2015) 57(12):1150–8. 10.1111/dmcn.12769 25907848 PMC4618264

[12] Isenberg JC Templer A Gao F Titus JB Gutmann DH. Attention skills in children with neurofibromatosis type 1. J Child Neurol (2013) 28(1):45–9. 10.1177/0883073812439435 22496119

[13] Lehtonen A Garg S Roberts SA Trump D Evans DG Green J Cognition in children with neurofibromatosis type 1: data from a population-based study. Dev Med Child Neurol (2015) 57(7):645–51. 10.1111/dmcn.12734 29927487

[14] Payne JM Barton B Shores EA North KN. Paired associate learning in children with neurofibromatosis type 1: implications for clinical trials. J Neurol (2013) 260(1):214–20. 10.1007/s00415-012-6620-5 22875098

[15] Michael GA Garcia S Herbillon V Lion-Francois L. Reactivity to visual signals in neurofibromatosis type 1: is everything ok? Neuropsychology (2014) 28(3):423–8. 10.1037/neu0000046 24274026

[16] Ribeiro MJ d’Almeida OC Ramos F Saraiva J Silva ED Castelo-Branco M Abnormal late visual responses and alpha oscillations in neurofibromatosis type 1: a link to visual and attention deficits. J Neurodev Disord (2014) 6:4 10.1186/1866-1955-6-4 24559228 PMC3944002

[17] Champion JA Rose KJ Payne JM Burns J North KN. Relationship between cognitive dysfunction, gait, and motor impairment in children and adolescents with neurofibromatosis type 1. Dev Med Child Neurol (2014) 56(5):468–74. 10.1111/dmcn.12361 24387687

[18] Debrabant J Plasschaert E Caeyenberghs K Vingerhoets G Legius E Janssens S Deficient motor timing in children with neurofibromatosis type 1. Res Dev Disabil (2014) 35(11):3131–8. 10.1016/j.ridd.2014.07.059 25145806

[19] Gilboa Y Josman N Fattal-Valevski A Toledano-Alhadef H Rosenblum S. Underlying mechanisms of writing difficulties among children with neurofibromatosis type 1. Res Dev Disabil (2014) 35(6):1310–6. 10.1016/j.ridd.2014.03.021 24691356

[20] Gilboa Y Rosenblum S Fattal-Valevski A Toledano-Alhadef H Rizzo AS Josman N. Using a virtual classroom environment to describe the attention deficits profile of children with neurofibromatosis type 1. Res Dev Disabil (2011) 32(6):2608–13. 10.1016/j.ridd.2011.06.014 21757320

[21] Galasso C Lo-Castro A Di Carlo L Pitzianti MB D’Agati E Curatolo P Planning deficit in children with neurofibromatosis type 1: a neurocognitive trait independent from attention-deficit hyperactivity disorder (ADHD)? J Child Neurol (2014) 29(10):1320–6. 10.1177/0883073813517001 24532810

[22] Payne JM Arnold SS Pride NA North KN. Does attention-deficit-hyperactivity disorder exacerbate executive dysfunction in children with neurofibromatosis type 1? Dev Med Child Neurol (2012) 54(10):898–904. 10.1111/j.1469-8749.2012.04357.x 22845611

[23] Gilboa Y Rosenblum S Fattal-Valevski A Toledano-Alhadef H Josman N. Is there a relationship between executive functions and academic success in children with neurofibromatosis type 1? Neuropsychol Rehabil (2014) 24(6):918–35. 10.1080/09602011.2014.920262 24875728

[24] Pride NA Payne JM North KN. The impact of ADHD on the cognitive and academic functioning of children with NF1. Dev Neuropsychol (2012) 37(7):590–600. 10.1080/87565641.2012.695831 23066937

[25] Lidzba K Granstrom S Lindenau J Mautner VF. The adverse influence of attention-deficit disorder with or without hyperactivity on cognition in neurofibromatosis type 1. Dev Med Child Neurol (2012) 54(10):892–7. 10.1111/j.1469-8749.2012.04377.x 22881119

[26] Roy A Barbarot S Roulin JL Charbonnier V Fasotti L Stalder JF Is executive function specifically impaired in children with neurofibromatosis type 1? A neuropsychological investigation of cognitive flexibility. Appl Neuropsychol Child (2014) 3(2):94–102. 10.1080/21622965.2012.704185 24716868

[27] Allen T Willard VW Anderson LM Hardy KK Bonner MJ. Social functioning and facial expression recognition in children with neurofibromatosis type 1. J Intellect Disabil Res (2016) 60(3):282–93. 10.1111/jir.12248 26805654

[28] Plasschaert E Descheemaeker MJ Van Eylen L Noens I Steyaert J Legius E. Prevalence of autism spectrum disorder symptoms in children with neurofibromatosis type 1. Am J Med Genet B Neuropsychiatr Genet (2015) 168B(1):72–80. 10.1002/ajmg.b.32280 25388972

[29] Garg S Plasschaert E Descheemaeker MJ Huson S Borghgraef M Vogels A Autism spectrum disorder profile in neurofibromatosis type I. J Autism Dev Disord (2015) 45(6):1649–57. 10.1007/s10803-014-2321-5 25475362

[30] Walsh KS Velez JI Kardel PG Imas DM Muenke M Packer RJ Symptomatology of autism spectrum disorder in a population with neurofibromatosis type 1. Dev Med Child Neurol (2013) 55(2):131–8. 10.1111/dmcn.12038 23163951

[31] Hyman SL Gill DS Shores EA Steinberg A Joy P Gibikote SV Natural history of cognitive deficits and their relationship to MRI T2-hyperintensities in NF1. Neurology (2003) 60(7):1139–45. 10.1212/01.WNL.0000055090.78351.C1 12682321

[32] Gill DS Hyman SL Steinberg A North KN. Age-related findings on MRI in neurofibromatosis type 1. Pediatr Radiol (2006) 36(10):1048–56. 10.1007/s00247-006-0267-2 16912896

[33] Payne JM Pickering T Porter M Oates EC Walia N Prelog K Longitudinal assessment of cognition and T2-hyperintensities in NF1: an 18-year study. Am J Med Genet A (2014) 164A(3):661–5. 10.1002/ajmg.a.36338 24357578

[34] Roy A Barbarot S Charbonnier V Gayet-Delacroix M Stalder JF Roulin JL Examining the frontal subcortical brain vulnerability hypothesis in children with neurofibromatosis type 1: are T2-weighted hyperintensities related to executive dysfunction? Neuropsychology (2015) 29(3):473–84. 10.1037/neu0000151 25365565

[35] Piscitelli O Digilio MC Capolino R Longo D Di Ciommo V Neurofibromatosis type 1 and cerebellar T2-hyperintensities: the relationship to cognitive functioning. Dev Med Child Neurol (2012) 54(1):49–51. 10.1111/j.1469-8749.2011.04139.x 22107256

[36] Aydin S Kurtcan S Alkan A Guler S Filiz M Yilmaz TF Relationship between the corpus callosum and neurocognitive disabilities in children with NF-1: diffusion tensor imaging features. Clin Imaging (2016) 40(6):1092–5. 10.1016/j.clinimag.2016.06.013 27423006

[37] Huijbregts SCJ Loitfelder M Rombouts SA Swaab H Verbist BM Arkink EB Cerebral volumetric abnormalities in neurofibromatosis type 1: associations with parent ratings of social and attention problems, executive dysfunction, and autistic mannerisms. J Neurodev Disord (2015) 7(1):1–9. 10.1186/s11689-015-9128-3 26473019 PMC4607002

[38] Loitfelder M Huijbregts SC Veer IM Swaab HS Van Buchem MA Schmidt R Functional connectivity changes and executive and social problems in neurofibromatosis type I. Brain Connect (2015) 5(5):312–20. 10.1089/brain.2014.0334 25705926 PMC4490703

[39] Violante IR Ribeiro MJ Cunha G Bernardino I Duarte JV Ramos F Abnormal brain activation in neurofibromatosis type 1: a link between visual processing and the default mode network. PLoS One (2012) 7(6):e38785. 10.1371/journal.pone.0038785 22723888 PMC3377684

[40] Li W Cui Y Kushner SA Brown RA Jentsch JD Frankland PW The HMG-CoA reductase inhibitor lovastatin reverses the learning and attention deficits in a mouse model of neurofibromatosis type 1. Curr Biol (2005) 15(21):1961–7. 10.1016/j.cub.2005.09.043 16271875

[41] Payne JM Barton B Ullrich NJ Cantor A Hearps SJ Cutter G Randomized placebo-controlled study of lovastatin in children with neurofibromatosis type 1. Neurology (2016) 87(24):2575–84. 10.1212/WNL.0000000000003435 27956565 PMC5207004

[42] van der Vaart T Plasschaert E Rietman AB Renard M Oostenbrink R Vogels A Simvastatin for cognitive deficits and behavioural problems in patients with neurofibromatosis type 1 (NF1-SIMCODA): a randomised, placebo-controlled trial. Lancet Neurol (2013) 12(11):1076–83. 10.1016/S1474-4422(13)70227-8 24090588

[43] Engert V Pruessner JC. Dopaminergic and noradrenergic contributions to functionality in ADHD: the role of methylphenidate. Curr Neuropharmacol (2008) 6(4):322–8. 10.2174/157015908787386069 19587853 PMC2701285

[44] Lidzba K Granstroem S Leark RA Kraegeloh-Mann I Mautner VF. Pharmacotherapy of attention deficit in neurofibromatosis type 1: effects on cognition. Neuropediatrics (2014) 45(4):240–6. 10.1055/s-0034-1368117 24504420

[45] Lion-Francois L Gueyffier F Mercier C Gerard D Herbillon V Kemlin I The effect of methylphenidate on neurofibromatosis type 1: a randomised, double-blind, placebo-controlled, crossover trial. Orphanet J Rare Dis (2014) 9:142. 10.1186/s13023-014-0142-4 25205361 PMC4172829

[46] Levine TM Materek A Abel J O’Donnell M Cutting LE Cognitive profile of neurofibromatosis type 1. Semin Pediatr Neurol (2006) 13(1):8–20. 10.1016/j.spen.2006.01.006 16818171

[47] Acosta MT Gioia GA Silva AJ. Neurofibromatosis type 1: new insights into neurocognitive issues. Curr Neurol Neurosci Rep (2006) 6(2):136–43. 10.1007/s11910-996-0036-5 16522267

[48] Klein-Tasman BP Janke KM Luo W Casnar CL Hunter SJ Tonsgard J Cognitive and psychosocial phenotype of young children with neurofibromatosis-1. J Int Neuropsychol Soc (2014) 20(1):88–98. 10.1017/S1355617713001227 24229851 PMC4249943

[49] Watt SE Shores A North KN. An examination of lexical and sublexical reading skills in children with neurofibromatosis type 1. Child Neuropsychol (2008) 14(5):401–18. 10.1080/09297040701595505 17963094

[50] Alivuotila L Hakokari J Visnapuu V Korpijaakko-Huuhka AM Aaltonen O Happonen RP Speech characteristics in neurofibromatosis type 1. Am J Med Genet A (2010) 152A(1):42–51. 10.1002/ajmg.a.33178 20034087

[51] Brei NG Klein-Tasman BP Schwarz GN Casnar CL. Language in young children with neurofibromatosis-1: relations to functional communication, attention, and social functioning. Res Dev Disabil (2014) 35(10):2495–504. 10.1016/j.ridd.2014.06.016 24995687

[52] Batista PB Lemos SM Rodrigues LO de Rezende NA. Auditory temporal processing deficits and language disorders in patients with neurofibromatosis type 1. J Commun Disord (2014) 48:18–26. 10.1016/j.jcomdis.2013.12.002 24447521

[53] Hyman SL Shores A North KN. The nature and frequency of cognitive deficits in children with neurofibromatosis type 1. Neurology (2005) 65(7):1037–44. 10.1212/01.wnl.0000179303.72345.ce 16217056

[54] Huijbregts S Swaab H de Sonneville L. Cognitive and motor control in neurofibromatosis type I: influence of maturation and hyperactivity-inattention. Dev Neuropsychol (2010) 35(6):737–51. 10.1080/87565641.2010.508670 21038163

[55] Iannuzzi S Albaret JM Chignac C Faure-Marie N Barry I Karsenty C Motor impairment in children with neurofibromatosis type 1: effect of the comorbidity with language disorders. Brain Dev (2016) 38(2):181–7. 10.1016/j.braindev.2015.08.001 26321374

[56] Janke KM Klein-Tasman BP Garwood MM Davies WH Trapane P Holman KS Relations between executive functioning and academic performance in adolescents with neurofibromatosis-1. J Dev Phys Disabil (2014) 26(4):431–50. 10.1007/s10882-014-9375-3

[57] Plasschaert E Van Eylen L Descheemaeker MJ Noens I Legius E Steyaert J. Executive functioning deficits in children with neurofibromatosis type 1: the influence of intellectual and social functioning. Am J Med Genet B Neuropsychiatr Genet (2016) 171B(3):348–62. 10.1002/ajmg.b.32414 26773288

[58] Casnar CL Janke KM van der Fluit F Brei NG Klein-Tasman BP. Relations between fine motor skill and parental report of attention in young children with neurofibromatosis type 1. J Clin Exp Neuropsychol (2014) 36(9):930–43. 10.1080/13803395.2014.957166 25284746

[59] Ejerskov C Lasgaard M Ostergaard JR. Teenagers and young adults with neurofibromatosis type 1 are more likely to experience loneliness than siblings without the illness. Acta Paediatr (2015) 104(6):604–9. 10.1111/apa.12946 25625197

[60] Johnson BA Sheng X Perry AS Stevenson DA. Activity and participation in children with neurofibromatosis type 1. Res Dev Disabil (2014) 36C:213–21. 10.1016/j.ridd.2014.10.004 25462482

[61] Pasini A Lo-Castro A Di Carlo L Pitzianti M Siracusano M Rosa C Detecting anxiety symptoms in children and youths with neurofibromatosis type I. Am J Med Genet B Neuropsychiatr Genet (2012) 159B(7):869–73. 10.1002/ajmg.b.32095 22911924

[62] Wolters PL Burns KM Martin S Baldwin A Dombi E Toledo-Tamula MA Pain interference in youth with neurofibromatosis type 1 and plexiform neurofibromas and relation to disease severity, social-emotional functioning, and quality of life. Am J Med Genet A (2015) 167A(9):2103–13. 10.1002/ajmg.a.37123 25976979 PMC8323589

[63] Licis AK Vallorani A Gao F Chen C Lenox J Yamada KA Prevalence of sleep disturbances in children with neurofibromatosis type 1. J Child Neurol (2013) 28(11):1400–5. 10.1177/0883073813500849 24065580 PMC3805763

[64] Martin S Wolters P Baldwin A Gillespie A Dombi E Walker K Social-emotional functioning of children and adolescents with neurofibromatosis type 1 and plexiform neurofibromas: relationships with cognitive, disease, and environmental variables. J Pediatr Psychol (2012) 37(7):713–24. 10.1093/jpepsy/jsr124 22353803 PMC3404452

[65] Constantino JN Zhang Y Holzhauer K Sant S Long K Vallorani A Distribution and within-family specificity of quantitative autistic traits in patients with neurofibromatosis type I. J Pediatr (2015) 167(3):621–6.e1. 10.1016/j.jpeds.2015.04.075 26051969 PMC4792262

[66] Garg S Lehtonen A Huson SM Emsley R Trump D Evans DG Autism and other psychiatric comorbidity in neurofibromatosis type 1: evidence from a population-based study. Dev Med Child Neurol (2013) 55(2):139–45. 10.1111/dmcn.12043 23163236

[67] Tinker J Carbone PS Viskochil D Mathiesen A Ma KN Stevenson DA. Screening children with neurofibromatosis type 1 for autism spectrum disorder. Am J Med Genet A (2014) 164A(7):1706–12. 10.1002/ajmg.a.36549 24715629

[68] Chabernaud C Sirinelli D Barbier C Cottier JP Sembely C Giraudeau B Thalamo-striatal T2-weighted hyperintensities (unidentified bright objects) correlate with cognitive impairments in neurofibromatosis type 1 during childhood. Dev Neuropsychol (2009) 34(6):736–48. 10.1080/87565640903265137 20183730

[69] Feldmann R Denecke J Grenzebach M Schuierer G Weglage J. Neurofibromatosis type 1: motor and cognitive function and T2-weighted MRI hyperintensities. Neurology (2003) 61(12):1725–8. 10.1212/01.WNL.0000098881.95854.5F 14694037

[70] Feldmann R Schuierer G Wessel A Neveling N Weglage J. Development of MRI T2 hyperintensities and cognitive functioning in patients with neurofibromatosis type 1. Acta Paediatr (2010) 99(11):1657–60. 10.1111/j.1651-2227.2010.01923.x 21039823

[71] Hyman SL Gill DS Shores EA Steinberg A North KN. T2 hyperintensities in children with neurofibromatosis type 1 and their relationship to cognitive functioning. J Neurol Neurosurg Psychiatry (2007) 78(10):1088–91. 10.1136/jnnp.2006.108134 17299016 PMC2117545

[72] Cohen R Steinberg T Kornreich L Aharoni S Halevy A Shuper A. Brain imaging findings and social/emotional problems in Israeli children with neurofibromatosis type 1. Eur J Pediatr (2015) 174(2):199–203. 10.1007/s00431-014-2366-7 25027832

[73] Bearden CE Hellemann GS Rosser T Montojo C Jonas R Enrique N A randomized placebo-controlled lovastatin trial for neurobehavioral function in neurofibromatosis I. Ann Clin Transl Neurol (2016) 3(4):266–79. 10.1002/acn3.288 27081657 PMC4818747

[74] Gilmore A Milne R. Methylphenidate in children with hyperactivity: review and cost-utility analysis. Pharmacoepidemiol Drug Saf (2001) 10(2):85–94. 10.1002/pds.564 11499858

[75] Schachter HM Pham B King J Langford S Moher D. How efficacious and safe is short-acting methylphenidate for the treatment of attention-deficit disorder in children and adolescents? A meta-analysis. CMAJ (2001) 165(11):1475–88. 11762571 PMC81663

[76] Dennis M Francis DJ Cirino PT Schachar R Barnes MA Fletcher JM. Why IQ is not a covariate in cognitive studies of neurodevelopmental disorders. J Int Neuropsychol Soc (2009) 15(3):331–43. 10.1017/S1355617709090481 19402919 PMC3075072

